# Tobacco Smoking: The Evidence from Prevention and Cessation

**DOI:** 10.1155/2014/894208

**Published:** 2014-12-31

**Authors:** Giuseppe La Torre, Amy Ferketich, Maria Caterina Grassi

**Affiliations:** ^1^Department of Public Health and Infectious Diseases, Sapienza University of Rome, Italy; ^2^Division of Epidemiology, The Ohio State University College of Public Health, USA; ^3^Department of Physiology and Pharmacology “V. Erspamer”, Sapienza University of Rome, Italy

The role of tobacco smoking as a cause of many diseases is now well established from the scientific point of view. It is recognized that tobacco consumption is the leading cause of preventable deaths in the majority of high-income nations and increasingly in low- and middle-income nations.

This special issue presents the latest evidence in the fields of both smoking prevention and cessation.

*Prevention*. R. Grazuleviciene et al. addressed the important issues of maternal smoking during pregnancy and second-hand tobacco smoke (SHS) exposure. These issues have been recognized as not only a serious threat to public health but also a significant cause of damage to the immune system of the fetuses. They found that both maternal smoking during pregnancy and SHS are risk factors for childhood wheezing and overweight.

China has the largest number of smokers and people exposed to SHS in the world (63.7% of nonsmokers are exposed to STS in their homes). P. Zheng et al. examined the prevalence of and factors associated with smoke-free home policies in Shanghai, as well as reasons for implementing such a policy. They highlight that smoke-free home policies are in place in approximately one-third of households that took part in the survey, and this type of policy was associated with personal smoking behavior and social factors. The findings of their work suggest the need to urgently promote smoke-free home policies through tobacco control programs in the most populated country in the world. In line with this, two factors seem most important to include in a tobacco control program: increasing citizens' knowledge and awareness of tobacco-related problems and encouraging medical doctors to promote smoking cessation among their patients. A. S. Abdullah and colleagues investigated factors associated with SHS exposure from parental smoking in Chinese families. Their interesting results demonstrate that some parents did not know about health consequences of smoking and effects of SHS exposure on children. On the other hand, few parents were asked by pediatricians about the child's exposure to SHS at home, even when the child's illness was related to smoking. The role of medical doctors in promoting healthy behaviours is crucial, and suggestions coming from pediatricians about smoke-free homes and parental quitting would be easily acceptable to parents and other household members.

Moreover, another key issue in this field is the ability of laws to enhance smoking prevention and to promote smoking cessation. M. R. Gualano et al. present data from Italy, where a more restrictive law was introduced in 2005. They found a constant decrease in tobacco consumption in Italy between 2001 and 2013, but no join point related to the introduction of the law banning smoking was present. According to the authors, these findings confirm that tobacco control programs must focus on other intervention activities, such as increasing the price of cigarettes, using graphic warnings on tobacco products [1], and including health promotion activities. In another paper focused on indoor smoking bans, T. Tabuchi et al. present the results of an observational study carried out in Japan that demonstrated that workplace smoking bans could reduce smoking among male workers and the husbands of female workers, compared with a partial smoking ban.

Discouraging smoking initiation and motivating adolescent smokers to quit is a difficult task. Family and school environments are fundamental settings where these actions could have the highest likelihood of success. However, data coming from the Italian HBSC surveys (2002–2010), while indicating that the prevalence of regular tobacco use remained stable among 15-year-olds, also showed that many adolescents are exposed to SHS smoke, have at least one parent who smokes, and have seen teachers and students smoking at school. Moreover, there is a lot to do in other setting as well, given that the survey indicated that the vast majority of adolescents had no trouble in buying cigarettes, even though the sale of tobacco products to minors is prohibited. In another setting (Georgia), C. J. Berg et al. indicated some interesting issues for tobacco control interventions among youths, underlying some factors that need to be taken into account, such as gender, use of alcohol, and marijuana, but also the lower perceived risk among young people.

There is a lot of evidence to suggest that smoking cessation training for medical students is very important for tackling tobacco smoking among general population. However, there is also evidence that in many countries the prevalence of smokers among health professionals is high [2, 3]. M. C. Grassi et al. demonstrate that even a brief intervention with medical students, via a lecture on nicotine dependence, is useful in the short term to significantly increase knowledge of nicotine and tobacco related issues. However, this intervention needs to be more comprehensive and cover different time periods in the medical student curriculum.

*Cessation*. Nicotine dependence has a high prevalence among individuals addicted to illicit drugs. The association between methamphetamine (MA) use and cigarette smoking was surveyed by Z. Wang et al. who carried out a study in Beijing and Guangdong, China, and found that most MA users are current smokers. Higher MA dose and ever-use of ketamine or alcohol were associated with a higher probability of high nicotine dependence, and this in turn was associated with significantly higher euphoria and stronger sexual impulse after using MA. This interesting work suggests that factors such as gender, marital status, and use of other psychoactive substances are key factors to be considered in providing health education on tobacco control and smoking cessation among MA users.

Determining the factors associated with smoking cessation success is a key issue in the modern public health arena. This question was addressed by Kaleta et al. who reported their experience in Romania. While they found that both gender and cohabitation with nonsmokers were the strongest predictors of cessation, different patterns of association are present among females and males. In fact, while among men they found an association between long-term quitting smoking and factors such as being economically active, being aged 40 and over, and having an awareness of smoking health consequences, among women cessation was mainly associated with initiating smoking at an older age.

Are there any personality characteristics that are associated with smoking? Positivity (POS), defined as a general disposition conducive to facing experiences with a positive outlook, was studied by Grassi and colleagues as a possible predictor of relapse after quitting smoking among Italian adults. Among people who attended a group counseling program for smoking cessation, POS was significantly and negatively associated with smoking status and with craving to smoke. The authors concluded that a better understanding of the biological correlates of POS could be useful in developing future cessation interventions.

*Giuseppe La Torre* *Amy Ferketich* *Maria Caterina Grassi*
